# Complete mitochondrial genome of a Korean endemic freshwater mussel *Nodularia breviconcha* (Bivalvia: Unionidae)

**DOI:** 10.1080/23802359.2020.1846473

**Published:** 2021-01-11

**Authors:** Yu Rim Kim, Hee-kyu Choi, Kyeong-Sik Cheon, Hyuk Je Lee

**Affiliations:** a Department of Biological Science, Sangji University, Wonju, South Korea; b Molecular Ecology and Evolution Laboratory, Department of Biological Science, Sangji University, Wonju, South Korea

**Keywords:** *Nodularia breviconcha*, complete mitochondrial genome, molecular phylogeny, Korean endemic freshwater mussels, bitterling fishes

## Abstract

Mitochondrial genome sequences were first determined and analyzed for a Korean endemic freshwater mussel *Nodularia breviconcha* (synonym *Nodularia douglasiae sinuolatus*; Unionidae, Unionida, Bivalvia). The complete mitochondrial genome was 15,741 bp in length, including 13 protein-coding genes (PCGs), 22 tRNA genes, and 2 rRNA genes. The overall GC content of mitochondrial genome for *N. breviconcha* was 34.3%. Phylogenetic analysis of 18 species within the family Unionidae suggested that *Nodularia douglasiae* is the most closely related to *N. breviconcha*. Our study will provide baseline, but important information for future research on ecological and genetic/genomic characteristics of this species.

Freshwater mussels belonging to the family Unionidae are composed of 621 species and are widely distributed worldwide (Bogan 2008; Lopes-Lima et al. 2014). *Nodularia breviconcha* (synonym: *Nodularia douglasiae sinuolatus*; class Bivalvia, order Unionida, family Unionidae) had been classified as a subspecies of *Nodularia douglasiae* (*N. douglasiae sinuolatus*) (von Martens 1905). However, the taxonomic status of *N. douglasiae sinuolatus* was recently revised as *N. breviconcha* (Lopes-Lima et al. 2020). *Nodularia breviconcha* is a widespread mussel primarily inhabiting the middle and upstream rivers in Korea; however, its geographic distribution has not yet been precisely determined (Kwon 1990). While its closely related species *N. douglasiae* is broadly distributed across Northeast Asia, including China, Japan and Korea, *N. breviconcha* is endemic to the Korean Peninsula (Kil 1976). In particular, these species play an important role in the reproduction and ecology of coexisting bittering fishes (subfamily Acheilognathinae), as they are largely used as spawning grounds for bittering fishes (Choi and Lee 2018, 2019). A recent collapse of freshwater mussel populations owing to increasing anthropogenic pressure likely entails the subsequent decrease in population sizes of bitterling fishes (Onikura et al. 2006; Kuwahara et al. 2017).

In this study, we first reported the complete mitochondrial genome (mitogenome) sequences of a Korean endemic species, *N. breviconcha* and determined its phylogenetic position in reference to other 17 species within the family Unionidae. The specimen of *N. breviconcha* used for this study (voucher number: HJ13869_UDS1252) was collected from the Han River system (Pandae-ri, Jijeong-myeon, Wonju-si, Gangwon-do province; 37°21′53.93″N, 127°49′6.88″E) and stored at Molecular Ecology and Evolution Laboratory, Department of Biological Science, Sangji University in Korea. Total genomic DNA was extracted using G-spin^TM^ Total DNA extraction kit (iNtRON, Seongnam, Korea). Paired-end sequencing for the mitogenome of *N. breviconcha* was performed on the Miseq (Illumina Inc., San Diego, CA) platform. We obtained 3,346,733 raw read pairs with a length of 301 bp. The assembly and annotation of the mitogenome were accomplished using the Geneious prime®2020.0.3 (Biomatters Ltd, Auckland, New Zealand). We also compared the obtained mitogenome of *N. breviconcha* with the previously published mitogenome database of *N. douglasiae* (MF314443), which was suggested to be the most closely related species (Cha et al. 2018). The transfer RNA (tRNA) genes were confirmed by comparing them with those of two related species (*N. douglasiae*; MF314443, *Unio delphinus*; NC_033854). A circular mitochondrial genome map was drawn using OGDRAW program (Greiner et al. 2019).

The assembled mitogenome of *N. breviconcha* (GenBank accession No: MT955592) showed a length of 15,741 bp and an overall GC content of 34.3%, and a total nucleotide composition of A − 38.6%, C − 22.5%, G − 11.8%, and T − 27.1%. It consists of 13 protein-coding genes (PCGs), 22 tRNA genes, and 2 ribosomal RNA (rRNA) genes. Two large noncoding intergenic spacers (IGS) were present between *trnE* and *ND2* (316 bp) and also between *ND5* and *trnQ* (290 bp). Structure of PCGs in *N. breviconcha* was identical to that in *N. douglasiae* (MF314443), meaning that the number and order of genes were the same between the two species.

Phylogenetic analyses were performed with nucleotide sequences of 13 PCGs and 2 rRNA genes (12,950 bp) using 16 Unionidae species (including the study species) and two outgroups (*Lepidodesma languilati* and *Sinohyriopsis schlegelii*). The sequences were aligned using MAFFT (Katoh et al. 2002) and phylogenetic trees were then constructed by maximum likelihood (ML) and maximum parsimony (MP) methods using MEGA X (Kumar et al. 2018) with 1000 bootstrap replications. The two independent phylogenetic trees yielded the same topology. *Nodularia breviconcha* formed a monophyly with *N. douglasiae* with a high support value (BS = 100) (Figure 1), supporting the hypothesis that they are sister taxa. This study will provide baseline, but important information for future research on ecology and genetics/genomics of this species.

**Figure 1.**
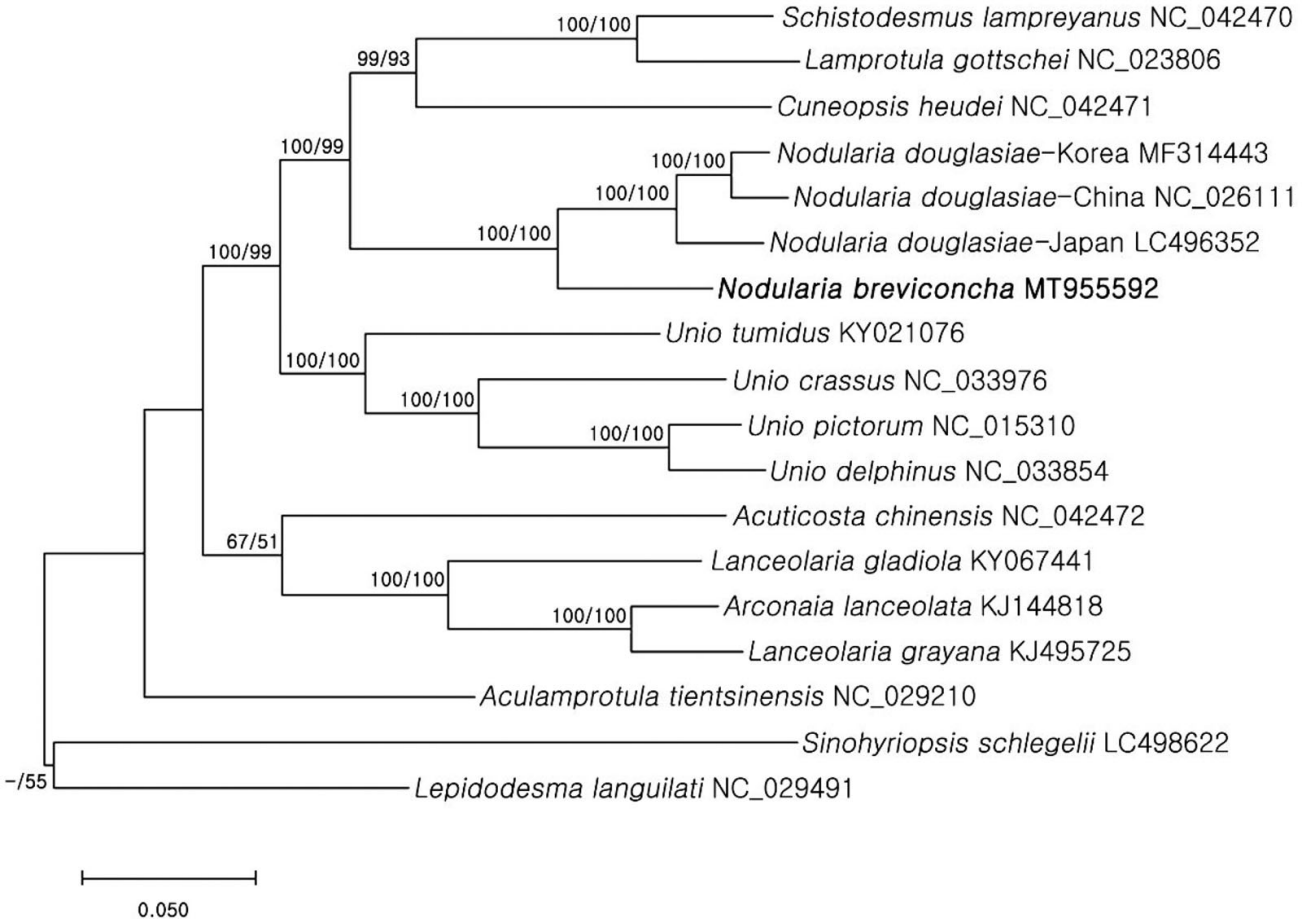
Molecular phylogenetic tree of 18 Unionidae species including Korean endemic *N. breviconcha*. Reconstruction of maximum likelihood (ML) and maximum parsimony (MP) trees was based on 13 PCGs and two rRNA genes (12,950 bp). Numbers at the branches represent the bootstrap support values for ML (left) and MP (right), respectively. Branching patterns and branch lengths follow the results of ML analysis.

## Data Availability

The data that support the findings of this study are openly available in GenBank of NCBI at http://www.ncbi.nlm.nih.gov, reference numbers MT955592 (http://www.ncbi.nlm.nih.gov/nuccore/MT955592) and PRJNA670559 (http://www.ncbi.nlm.nih.gov/bioproject/PRJNA670559).
